# Chemical Composition of *Pterospermum heterophyllum* Root and its Anti-Arthritis Effect on Adjuvant-Induced Arthritis in Rats *via* Modulation of Inflammatory Responses

**DOI:** 10.3389/fphar.2020.584849

**Published:** 2020-12-11

**Authors:** Li Yang, Ronghua Liu, Aiguo Fan, Jingjing Zhao, Yong Zhang, Junwei He

**Affiliations:** ^1^ Jiangxi University of Traditional Chinese Medicine, Nanchang, China; ^2^ Department of Pharmacy, Guangdong Women and Children Hospital, Guangzhou, China

**Keywords:** *Pterospermum heterophyllum* root, rheumatoid arthritis, chemical composition, flavonoid, inflammatory response

## Abstract

Rheumatoid arthritis (RA) is a chronic autoimmune inflammatory disease without effective and beneficial drugs. Many traditional folk medicines have been proven to be effective in treating RA. Among these, the root of *Pterospermum heterophyllum* Hance has been widely used as a traditional remedy against RA in China, but there is no scientific basis yet. The aim of this study was to investigate for the first time the chemical compositions and therapeutic effect of *P. heterophyllum* on adjuvant-induced arthritis (AIA) model in rats. 73 compounds were identified from *P. heterophyllum* based on ultra-performance liquid chromatography-quadrupole time-of-flight tandem mass spectrometry (UPLC-qTOF-MS/MS), and flavonoids may be partly responsible for the major anti-arthritic effect. In parallel, the *P. heterophyllum* extract at 160, 320, and 640 mg/kg/day were orally administered to rats for 22 days after post-administration adjuvant. The results showed that *P. heterophyllum* remarkably ameliorated histological lesions of the knee joint, increased body weight growth, decreased arthritis score, reduced thymus and spleen indices in model rats. Moreover, *P. heterophyllum* treatment persuasively downregulated the levels of rheumatoid factor (RF), C-reactive protein (CRP), tumor necrosis factor alpha (TNF-α), interleukin-1β (IL-1β), IL-6, IL-17, cyclooxygenase-2 (COX-2), 5-lipoxygenase (5-LOX) and matrix metalloproteinase-2 (MMP-2), and observably upregulated IL-4 and IL-10 levels in model rats. These findings suggest that *P. heterophyllum* has a prominent anti-RA effect on AIA rats by modulating the inflammatory responses, and supports the traditional folk use of this plant.

## Introduction

Rheumatoid arthritis (RA) is a chronic, systematic and autoimmune inflammatory disease that results in progressive synovitis, joint swelling and damage, synovial hyperplasia, and bone and cartilage erosion (Yousefi et al., 2014; Saleem et al., 2020; Zhu et al., 2020). Although the etiology of RA is intricate and vague, inflammatory factors, including pro-inflammatory cytokines, anti-inflammatory cytokines and inflammatory mediators, are responsible for bone and cartilage erosions, and play a crucial role in this disease (Wang et al., 2017a; Rui et al., 2019; Saleem et al., 2020). Additionally, the serum levels of these inflammatory mediators were determined by enzyme-linked immunosorbent assay (ELISA) kits (Lin et al., 2013; Wang et al., 2017a). Currently, immunosuppressants, biological agents and disease-modifying anti-rheumatic drugs (DMARDs), and steroidal and non-steroidal anti-inflammatory drugs (NSAIDs) are commonly used for the treatment of RA, but most of them display long-term adverse effects, toxicity and comorbidities (Dai et al., 2020; Li et al., 2019a, Li et al., 2019b; Rui et al., 2019). As a result of this, exploring effective and safe anti-RA drug candidates from natural products, especially traditional folk medicines, could be a momentous breakthrough.

Traditional Chinese medicines (TCMs) are decisive complementary and alternative medicines, which have been verified to be effective treating RA for centuries with more safety and little side-effects in China and other Southeast Asian countries (Bao et al., 2018; Li et al., 2019a, Li et al., 2019b; Lin et al., 2013; Jing et al., 2019; Yang et al., 2020). *Pterospermum heterophyllum* Hance is native only to China and widely distributed in Fujian, Guangdong, Guangxi and Hainan provinces, belonging to the Sterculiaceae family (Editorial Committee of Traditional Chinese Medicine 1999; Yang et al., 2016, 2019a). The root of *P. heterophyllum* is a vital TCM and has been used for centuries as an empiric treatment for RA and other inflammation-related diseases (Editorial Committee of Traditional Chinese Medicine 1999; Yang et al., 2016; Yang et al., 2019a). Despite good clinical practice and good clinical effects, the phytochemical profiling and anti-RA efficacy of *P. heterophyllum* are still unknown, leading to numerous obstacles in the clinical application and reasonable development of this plant.

Therefore, in this study, the AIA rat model was adopted to evaluate the therapeutic efficacy and underlying mechanisms of *P. heterophyllum*. Following this step, ultra-performance liquid chromatography-quadrupole time-of-flight tandem mass spectrometry (UPLC-qTOF-MS/MS) analysis was performed to explore the phytochemicals present in this plant. Our findings will provide adequate scientific evidence for the development and clinical application of *P. heterophyllum*.

## Materials and Methods

### Chemicals and Reagents

Pentobarbital sodium (Shanghai Rongbai Biological Technology Co., Ltd., Shanghai, China), Complete Freund’s adjuvant (CFA) and Histopaque 1,083 (Sigma Co., USA), MTX (Shanghai Xingyi Pharmaceutical Co., China), TNF-α, IL-1β, IL-4, IL-6, IL-10, IL-17, COX-2, 5-LOX and MMP-2 ELISA kits (Chuzhou Shinuoda Biological Technology Co., China) were used in this experiment.

### Plant Material and Extracts Preparation

Plant materials of *P. heterophyllum* roots were collected from the town of Pulu, Lipu Country, Guilin City, Guangxi, China (GPS location: 110.51682262,911,989, 24.576018798,987,043), in October 2017, and was authenticated by professor Ronghua Liu. A voucher specimen (No. PH20171024) for *P. heterophyllum* root was deposited in the author’s laboratory.

The dried and powdered roots of *P. heterophyllum* (1.0 kg) were extracted with 95% EtOH (5 L × 3) and subsequently with 50% EtOH (5 L × 3) by maceration at room temperature for 7 days. The ethanol crude extract of *P. heterophyllum* roots was filtrated and evaporated to obtain a black residue (PH, 160 g), with a yield of 16.0%.

According to the TCM clinical practice (9–30 g/day) (Editorial Committee of Traditional Chinese Medicine 1999), the dosage of *P. heterophyllum* roots for rat was 0.8–2.7 g/kg/day (body weight). Thus, the dosages of PH for rat were 1.0 g/kg (equivalent to 160 mg/kg crude extract, low-dose), 2.0 g/kg (320 mg/kg, medium-dose) and 4.0 g/kg (640 mg/kg, high-dose) in this experiment. All these extracts were dissolved in 0.3% sodium carboxymethyl cellulose (CMC-Na) for oral administration.

### Ultra-Performance Liquid Chromatography-Quadrupole Time-of-Flight Tandem Mass Spectrometry Analysis for Chemical Profiling

The identification of phytochemicals in the ethanol crude extract of PH was carried out using UPLC-qTOF-MS/MS in a Shimadzu UHPLC System (Kyoto, Japan) coupled with an AB SCIEX Triple TOF™ 5600 + system (Foster City, CA, USA) (Yang et al., 2019b). The chromatographic separation was conducted in an ACQUITY UPLC^®^BEH C_18_ (100 × 2.1 mm, 1.7 μm) maintained at 35°C. 0.1% aqueous formic acid (v/v, A) and acetonitrile (B) were used as mobile phases. The gradient elution with the flow rate of 0.3 ml/min was performed as follows: 0–8 min 5–8% B; 8–12 min 8–8% B; 12–17 min 8–12% B; 17–28 min 12–35% B; 28–35 min 55–55% B; 35–45 min 55–95% B; 45–47 min 95–95% B; 47–47.1 min 95–5% B; 47.1–50.0 min 5–5% B. The sample inject volume was 3 μL.

### Experimental Animals

Sprague-Dawley rats (weighing 160–180 g) were obtained from Beijing Vital River Laboratory Animal Technology Co., Ltd. (Beijing, China) and housed in cages at a room temperature of 21–25°C with 12 light/dark reverse cycles.

### Experimental Design

**FIGURE 1:**
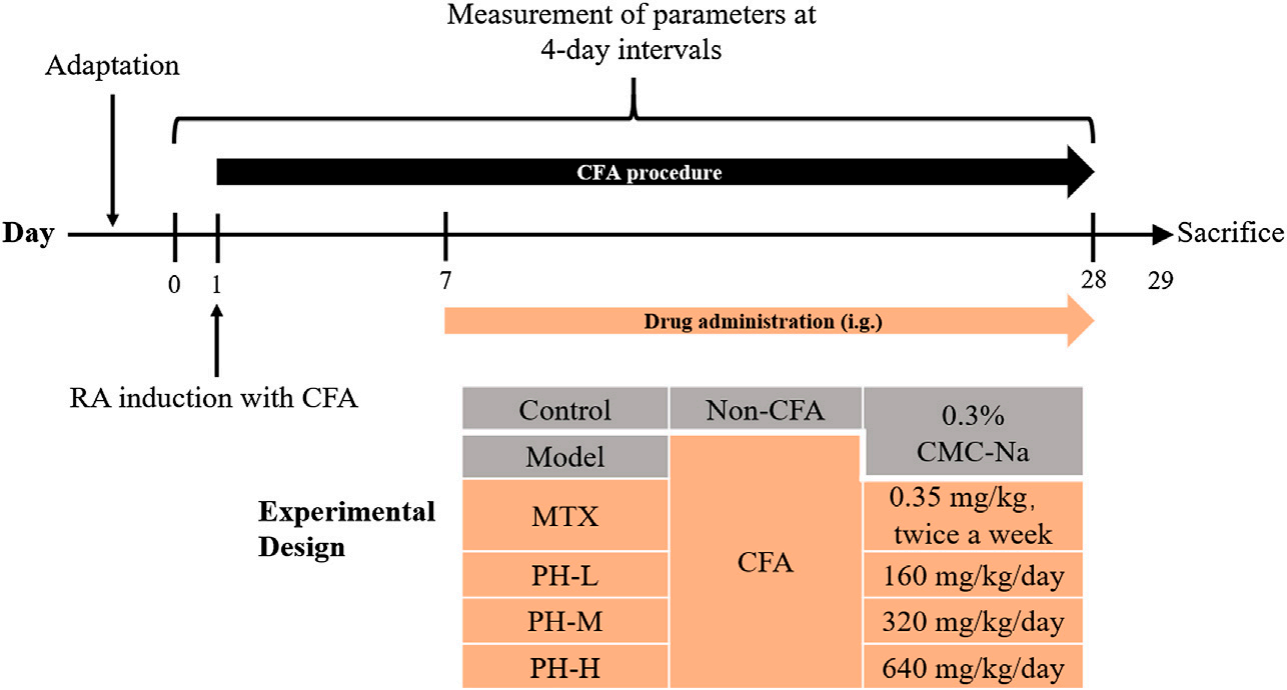
The experimental schedule of *P. heterophyllum* in the AIA rats.

After adaptive feeding, the rats were randomly assigned to six groups (n = 8): normal control (Control), AIA model (AIA), AIA model + MTX (AIA + MTX, 0.35 mg/kg), AIA model + PH low-dose (AIA + PH-L, 160 mg/kg), AIA model + PH medium-dose (AIA + PH-M, 320 mg/kg), and AIA model + PH high-dose (AIA + PH-H, 640 mg/kg). In accordance with the previous method, the AIA rat model was induced by a single intradermal injection of 100 μL CFA into the rat’s left hind footpad (day 1) (Yang et al., 2016; Pan et al., 2017). After the establishment of the AIA model, all PH crude extracts were administered orally once a day from day 7 to day 28. MTX was used as a positive drug and administered intragastrically (i.g.) twice a week. Meanwhile, the rats in the normal control group and the AIA model group were treated with an equal volume of 0.3% CMC-Na. The experimental protocol of PH effect on CFA-induced RA in rats was shown in Figure 1.

### Evaluation of Rheumatoid Arthritis

The body weight and arthritis score of rats were measured every 4 days. The arthritis scores of rat paws were evaluated using a 5-point scale (Yang et al., 2016; Jing et al., 2019): 0 = no erythema or swelling; 1 = erythema or toe joints swelling; 2 = toes and joints swelling; 3 = toes swelling and ankle joints swelling; 4 = the entire paw swelling and ankle joints swelling. The maximum arthritis score of each rat was set at 16 (4 points ×4 paws).

On day 29th day after immunization, all rats were killed after anesthesia (1% pentobarbital sodium, 40 mg/kg), and the immune organs including thymus and spleen were harvested and weighed. The index of thymus or spleen (mg/g) = thymus or spleen wet weight/body weight (Lin et al., 2013; Jing et al., 2019).

### Biochemical and Hematological Analysis

Blood was collected from the carotid artery of rats after being anesthetized. The peripheral blood mononuclear cells (PBMC) isolation process was performed according to the previous method (Lin et al., 2013). The serum levels of RF, CRP, TNF-α, IL-1β, IL-4, IL-6, IL-10, and IL-17, and the PBMC levels of COX-2, 5-LOX and MMP-2, were quantified by commercially available ELISA kits based on the manufacturer’s instructions (Chuzhou Shinuoda Biological Technology Co., China).

### Histopathological Examination

The ankle joints of the rats were removed and fixed in 4% (w/v) paraformaldehyde, decalcified in 10% ethylene-diamine-tetraacetic acid (EDTA) at 4°C for 30 days. Tissues were embedded in paraffin and 4 µm joint sections were obtained. Subsequently, the sections were deparaffinized, dehydrated and stained with hematoxylin and eosin (HE). These sections were examined with a DS-F12 microscope (magnification, ×100, Nikon Corporation, Japan) for histopathological analysis.

### Statistical Analysis

Graphpad Prism6 was used for statistical analysis, and the data were presented as the means ± standard deviation (SD). One-way analysis of variance (ANOVA) and Tukey’s test were used for comparison differences groups. Differences with *p* < 0.05 indicated statistical significance.

### Ethics Statement

All the experiments were carried out in adherence with the guidelines of the Institutional Animal Care and Use Committee of China and were approved by the Animal Care and Research Committee of Jiangxi University of Traditional Chinese Medicine. All surgical procedures were performed under sodium pentobarbital anesthesia to minimize suffering.

## Results

### Phytochemicals Identification of *P. heterophyllum* Using Ultra-Performance Liquid Chromatography-Quadrupole Time-of-Flight Tandem Mass Spectrometry

**FIGURE 2:**
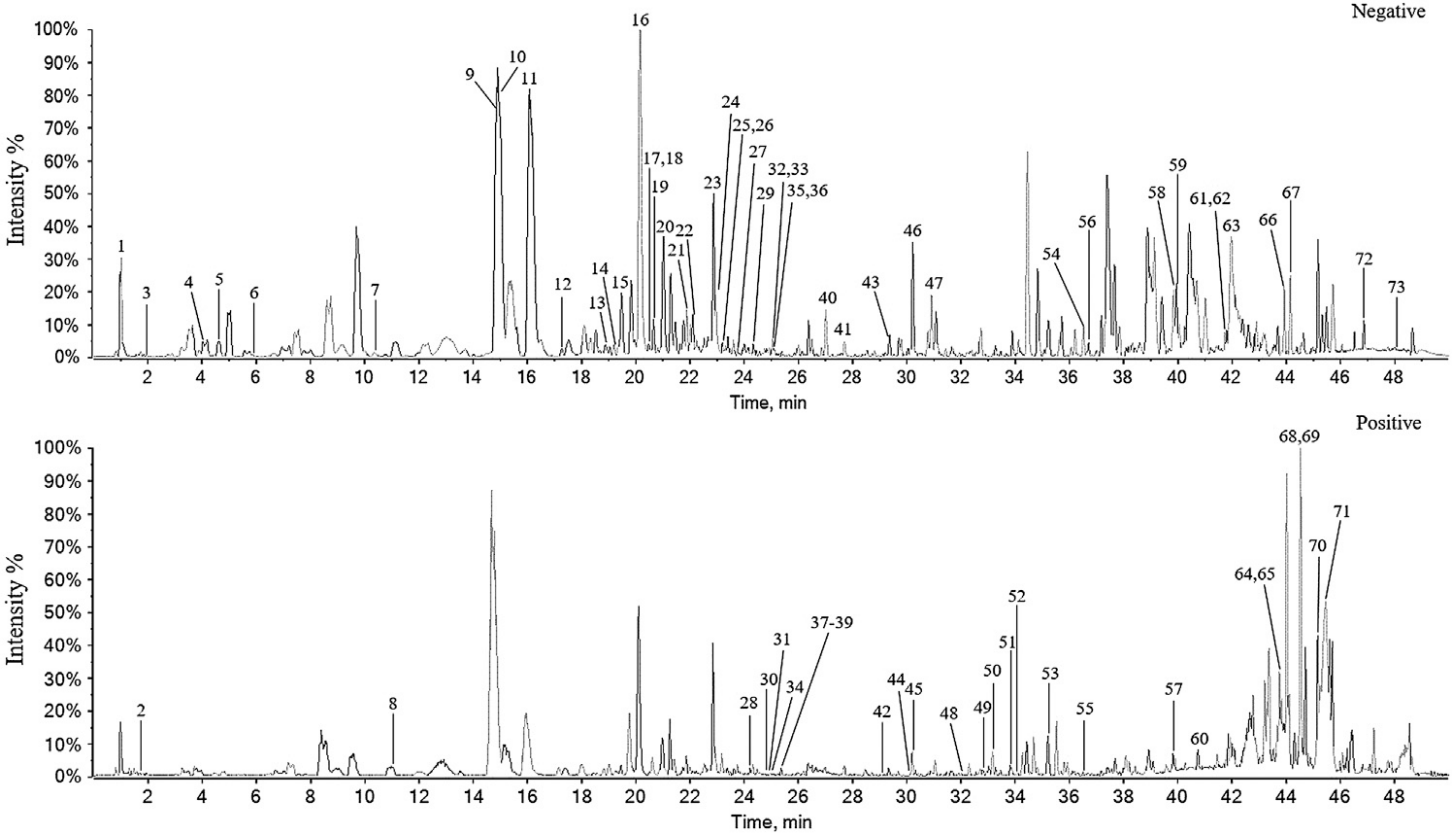
The base peak chromatogram of *P. heterophyllum* by UPLC-Q-TOF-MS/MS in negative and positive-ion modes.

The chemical constituents corresponding to the chromatographic peaks were determined by MS/MS analysis using negative- and positive-ion modes based on literature and databases (Yang et al., 2019b) (Figure 2).

As a result, a total of 73 compounds, including 34 flavonoids, eight fatty acids, seven triterpenoids, six steroids, six alkaloids, five phenylpropanoids, and seven others were identified from *P. heterophyllum* based on UPLC-qTOF-MS/MS (Table 1). Therefore, this study has greatly enriched the chemical constituents and diversity. Among them, 15 flavonoids, including procyanidin B2 (peak 9) (Wang et al., 2017b), dihydromyricetin (peak 10) (Chu et al., 2018), (-)-epicatechin (peak 11) (Osman et al., 2019), puerarin (peak 12) (Wang et al., 2016), rutin (peak 24) (Sun et al., 2017), naringin (peak 28) (Ahmad et al., 2014), hesperidin (peak 32) (Li et al., 2019a), myricetin (peak 35) (Yuan et al., 2015), eriodictyol (peak 40) (Lei et al., 2020), quercetin (peak 41) (Saccol et al., 2019), naringenin (peak 43) (Zhu et al., 2015), kaempferol (peak 44) (Pan et al., 2018), diosmetin (peak 46) (Chen et al., 2019), nobiletin (peak 51) (Li et al., 2019a), and tangeretin (peak 53) (Li et al., 2019c), have been reported to have arthritis inhibitory effect in rats. Additionally, cinnamaldehyde (peak 8, phenylpropanoid) (Mateen et al., 2019), ursolic acid (peak 65, triterpenoid) (Kim et al., 2018), linoleic acid (peak 67, fatty acid) (Wang et al., 2011) and emodin (peak 54, other) (Zhu et al., 2013) were also exhibited anti-arthritis activities *in vivo*. Consequently, flavonoids may be responsible for the major active constituents in the roots of *P. heterophyllum* against RA.

**TABLE 1:** Chemical constituents of *P. heterophyllum* identified by UPLC-qTOF-MS/MS in negative- and positive-ion modes.

No	*t*_R_ (min)	Ion mode	Molecular weight	Measured mass	Error (ppm)	Formula	Fragments	Identification	Type
1	1.01	[M–H]^−^	342.11621	341.10944	1.5	C_12_H_22_O_11_	341.1091,179.0589,161.0489,119.0400,113.0295	Sucrose	Other
2	1.71	[M + H]^+^	267.09675	268.10428	0.9	C_10_H_13_N_5_O_4_	136.0613,119.0343	Adenosine	Alkaloid
3	1.98	[M–H]^−^	283.09167	282.0853	3.2	C_10_H_13_N_5_O_5_	150.0451,133.0200,108.0287	Guanosine	Alkaloid
4	4.09	[M–H]^−^	316.07943	315.07218	0.1	C_13_H_16_O_9_	153.0218,109.0346	Protocatechuic acid-hexoside	Other
5	4.38	[M–H]^−^	330.09508	329.0879	0.3	C_14_H_18_O_9_	167.0369,152.0134,133.0353,123.0495,108.0259	Phaseoloidin	Phenylpropanoid
6	5.87	[M–H]^−^	360.10565	359.09827	-0.3	C_15_H_20_O_10_	197.0470, 182.0245,167.0008,153.0603,138.0366,123.0128	Methoxypolygoacetophenoside	Other
7	10.36	[M–H]^−^	306.07395	305.06722	1.8	C_15_H_14_O_7_	305.0599,287.0550,269.0455,243.0298,225.0553,201.0592,164.0125,161.0262,137.0256,130.9705,125.0273,121.0311,109.0347	(-)-Eplgallocatechin	Flavonoid
8	11.15	[M + H]^+^	132.05751	133.06506	2	C_9_H_8_O	105.0696,103.0551	Cinnamaldehyde	Phenylpropanoid
9	14.93	[M–H]^−^	578.14243	577.13328	−3.2	C_30_H_26_O_12_	577.1320,451.1019,425.0859,407.0758,289.0718,245.0823,161.0265,125.0277	Procyanidin B2	Flavonoid
10	15.01	[M–H]^−^	320.05322	319.04616	0.7	C_15_H_12_O_8_	193.0153,191.0347,161.0227,151.0060,137.0269,125.0268	Dihydromyricetin	Flavonoid
11	16.12	[M–H]^−^	290.07904	289.07286	3.8	C_15_H_14_O_6_	289.0721,245.0833,221.0836,205.0518,203.0733,125.0287,123.0491,109.0340	(-)-Epicatechin	Flavonoid
12	17.27	[M–H]^−^	416.11073	415.10294	−1.3	C_21_H_20_O_9_	415.1047,307.0607,295.0621,277.0515,267.0671,253.0519	Puerarin	Flavonoid
13	18.84	[M–H]^−^	446.1213	445.11343	−1.3	C_22_H_22_O_10_	445.1113,325.0720,310.0493,297.0781,282.0549	3′-methoxypuerarin	Flavonoid
14	19.05	[M–H]^−^	522.21011	521.20164	−2.3	C_26_H_34_O_11_	359.1508，344.1279,313.1090,241.0523	Urolignoside	Phenylpropanoid
15	19.67	[M–H]^−^	548.15299	547.14409	−3	C_26_H_28_O_13_	547.1443,295.0624,277.0529,267.0678	Mirificin	Flavonoid
16	20.16	[M–H]^−^	866.20582	865.19658	−2.3	C_45_H_38_O_18_	865.1956,847.1881,739.1656,713.1496,695.1387,577.1328,575.1178,451.1030,425.0877,407.0768,287.0569,243.0317	B-type procyanidin trimer	Flavonoid
17	20.40	[M–H]^−^	594.13734	593.12836	−2.9	C_30_H_26_O_13_	593.1302,575.1200,557.1046,467.0988,423.0719,405.0596,387.0524,313.0353,305.0671,287.0568,243.0316,195.0295,161.0267,125.0281	Proanthocyanidins dimer	Flavonoid
18	20.47	[M–H]^−^	438.11621	437.1081	−1.9	C_20_H_22_O_11_	437.1076,311.0772,297.0618,269.0698,167.0362,149.0269,125.0277	Loquatoside	Flavonoid
19	20.72	[M–H]^−^	882.20073	881.19123	−2.5	C_45_H_38_O_19_	881.1929,863.1863,755.1616,745.1931,729.1377,711.1371,593.1273,575.1173,467.0996,423.0719,305.0674,287.0577,243.0293,125.0260	Proanthocyanidins trimer	Flavonoid
20	21.02	[M–H]^−^	1,154.692	1,153.2568	−4.4	C_60_H_50_O_24_	1,153.2545,865.1957,739.1650,575.1177,449.0873,287.0573	B-type procyanidin tetramer	Flavonoid
21	21.88	[M–H]^−^	482.14243	481.1338	−2.8	C_22_H_26_O_12_	481.1326,357.1341,311.0779,297.0618,168.0446,167.0368,154.0296,153.0210,108.0243	Heterophylloside B	Other
22	22.06	[M + H]^+^	304.0583	305.06576	0.6	C_15_H_12_O_7_	287.0529,231.0641,213.0530,153.0178	Dihydroquercetin	Flavonoid
23	22.69	[M–H]^−^	464.09548	463.08696	-2.7	C_21_H_20_O_12_	463.0886,316.0212,301.0367,300.0290,151.0074	Hyperin	Flavonoid
24	22.71	[M–H]^−^	610.15339	609.14485	−2.1	C_27_H_30_O_16_	609.1452,301.0360,300.0288,271.0241,255.0296	Rutin	Flavonoid
25	23.20	[M–H]^−^	576.12678	575.1178	−3	C_30_H_24_O_12_	575.1203,557.1119,539.0974,449.0889,423.0716,407.0776,289.0726,285.015,245.0831	Procyanidin A2	Flavonoid
26	23.33	[M–H]^−^	864.19016	863.18099	−2.2	C_45_H_36_O_18_	863.1832,711.1340,693.1239,575.1184,539.0976,449.0871,423.0724,285.0419	A-type procyanidin trimer	Flavonoid
27	23.85	[M–H]^−^	594.15847	593.14921	−3.3	C_27_H_30_O_15_	593.1485,285.0411,284.0330,255.0312	Kaempferol-3-O-[2-rhamnose (1–2)]-glucopyranoside	Flavonoid
28	24.12	[M + H]^+^	580.17921	581.18707	1	C_27_H_32_O_14_	581.2823,417.1038,315.0841,273.0740,219.0270,153.0175,129.0538	Naringin	Flavonoid
29	24.4	[M–H]^−^	448.10056	447.09232	−2.2	C_21_H_20_O_11_	447.0945,301.0359,285.0408,271.0266,257.0490,255.0323,229.0536,151.0046	Kaempferol-7-O-D-glucoside	Flavonoid
30	24.85	[M + COOH]^-^	678.50438	723.50142	−0.1	C_36_H_66_N_6_O_6_	723.5011,677.4934,419.0733,225.1590	cyclo hexaleucyl (isoleucyl)	Alkaloid
31	24.92	[M + H]^+^	302.07904	303.08638	0.2	C_16_H_14_O_6_	303.0843,177.0552,153.0172,145.0283,137.0585,117.0329	Hesperetin	Flavonoid
32	24.94	[M–H]^−^	610.18977	609.18109	−2.3	C_28_H_34_O_15_	609.1807,489.1382,343.0826,301.0722,286.0493	Hesperidin	Flavonoid
33	24.97	[M–H]^-^	462.11621	461.10857	−0.8	C_22_H_22_O_11_	461.1071,446.0864,298.0483,283.0272,255.0309	Chrysoeriol-7-O-galactoside	Flavonoid
34	25.09	[M + H]^+^	274.08412	275.09143	0.1	C_15_H_14_O_5_	169.0490,107.0490	Phloretin	Other
35	25.10	[M–H]^−^	318.03757	317.03072	1.4	C_15_H_10_O_8_	317.0319,289.0733,258.0553,207.0678,192.0442,178.9972,152.0151,151.0075,125.0286,109.0331	Myricetin	Flavonoid
36	25.12	[M–H]^−^	436.13695	435.12907	−1.4	C_21_H_24_O_10_	273.0775,179.0355,167.0366,125.0267,123.0482,119.0527	Phloridzin	Other
37	25.62	[M + COOH]^-^	791.58845	836.58353	−2.4	C_42_H_77_N_7_O_7_	836.5830,790.5797	cyclo heptaleucyl (isoleucyl)	Alkaloid
38	26.26	[M + COOH]^-^	904.67251	949.66779	−1.9	C_48_H_88_N_8_O_8_	949.6680,903.6638	cyclo octaleucyl (isoleucyl)	Alkaloid
39	26.78	[M + COOH]^-^	1,017.75658	1,062.7511	−2.5	C_54_H_99_N_9_O_9_	1,062.7547,1016.7439	cyclo nonaleucyl (isoleucyl)	Alkaloid
40	27.01	[M–H]^−^	288.06339	287.05721	3.8	C_15_H_12_O_6_	151.0078,135.0492,134.0409,107.0191	Eriodictyol	Flavonoid
41	27.70	[M–H]^−^	302.04265	301.03621	2.8	C_15_H_10_O_7_	301.0357,273.0418,245.0464,179.0002,151.0067,121.0331	Quercetin	Flavonoid
42	29.19	[M + H]^+^	386.13655	387.14419	0.9	C_21_H_22_O_7_	387.1431,357.1300,191.0687,181.0491,163.0741,137.0587	Kushenol W	Flavonoid
43	29.36	[M–H]^−^	272.06847	271.06247	4.7	C_15_H_12_O_5_	271.0618,187.0418,151.0063,119.0541	Naringenin	Flavonoid
44	30.08	[M + H]^+^	286.04774	287.0549	−0.4	C_15_H_10_O_6_	287.0531, 258.0480,213.0528,153.0141	Kaempferol	Flavonoid
45	30.21	[M + H]^+^	488.35018	489.35729	−0.3	C_30_H_48_O_5_	453.3349,435.3228,425.3228,407.3288,205.1577	Trihydroxy-urs-12-en-28-oic acid	Triterpenoids
46	30.27	[M–H]^−^	300.06339	299.05697	2.9	C_16_H_12_O_6_	299.0566,284.0340,256.0396,227.0370	Diosmetin	Flavonoid
47	30.91	[M–H]^−^	330.24062	329.23415	2.4	C_18_H_34_O_5_	329.2344,311.2236,293.2138,229.1463,211.1364,183.1420,171.1059,139.1173	Trihydroxy-octadecaenoic acid	Fatty acids
48	32.10	[M + H]^+^	260.10486	261.1119	−0.9	C_15_H_16_O_4_	243.1019,213.0533,189.0533,187.0393,159.0432,131.0484,103.0539	Meranzin	Phenylpropanoid
49	32.79	[M + H]^+^	342.11034	343.1179	0.8	C_19_H_18_O_6_	343.1151,328.0910,313.0691,285.0751,181.0113,153.0186	5,7,2′,3′-tetramethoxyflavone	Flavonoid
50	33.30	[M + COOH]^-^	740.43469	785.42943	−3	C_39_H_64_O_13_	739.4244,577.3648	20 (22)-en-5β-furost-3β,15β-diol-3-*O*-β-d-glucopyranosyl-(1→2)-β-d-galactopyranoside	Steroid
51	33.88	[M + H]^+^	402.13147	403.13911	0.9	C_21_H_22_O_8_	403.1384,388.1158,373.0912,327.0850,183.0273	Nobiletin	Flavonoid
52	34.01	[M + H]^+^	344.0896	345.09727	1.1	C_18_H_16_O_7_	345.0955,330.0707, 315.0534,287.0523,281.0426,181.0426	Santin	Flavonoid
53	35.24	[M + H]^+^	372.1209	373.12858	1.1	C_20_H_20_O_7_	373.1286,358.1045,343.0810,325.0700,312.0994,297.0748	Tangeretin	Flavonoid
54	36.43	[M–H]^−-^	270.05282	269.04621	2.5	C_15_H_10_O_5_	269.0459,241.0511,225.0575,213.0602	Emodin	Other
55	36.44	[M + H]^+^	202.02661	203.03372	−0.8	C_11_H_6_O_4_	203.0334,175.0511,159.0434,147.0438,131.0459,129.0335,119.0508	Xanthotoxol	Phenylpropanoid
56	36.52	[M–H]^−-^	314.24571	313.2389	1.5	C_18_H_34_O_4_	313.2389,295.2283,277.2172,201.1150,171.1049	Dihydroxy-octadecaenoic acid	Fatty acids
57	39.83	[M + H]^+^	472.35526	473.36283	0.6	C_30_H_48_O_4_	437.3402,409.3445,391.3338,205.1565,203.1769,189.1616	Maslinic acid	Triterpenoids
58	39.84	[M–H]^−^	518.36074	517.35111	−4.6	C_31_H_50_O_6_	471.3456	(1,3,9)-24-hydroperoxy-1,3-dihydroxy-5-methyl-9,19-cyclolanost-25-en-28-oic acid	Triterpenoids
59	40.29	[M–H]^−^	294.2195	293.21285	2.1	C_18_H_30_O_3_	293.2125,197.1216,185.1200,125.0991	Hydroxy-octadecatrienoic acid	Fatty acids
60	41.46	[M + H]^+^	352.26136	353.26885	0.6	C_21_H_36_O_4_	353.2658,261.2203,243.2099,173.1313,135.1160,121.1007,107.0854	Pregnane-3,11,17,20-tetrol	Steroid
61	41.75	[M–H]^−^	312.30283	311.2958	0.8	C_20_H_40_O_2_	311.2597,293.2483,275.2358,171.1047	Arachidic acid	Fatty acids
62	41.80	[M–H]^−^	296.23515	295.22824	1.3	C_18_H_32_O_3_	295.2290,277.2197,251.2395,221.1934,169.1618	Hydroxy-octadecadienoic acid	Fatty acids
63	42.12	[M–H]^−^	312.23006	311.22319	1.3	C_18_H_32_O_4_	311.2230,171.1051,155.1469,127.1163,111.0860,109.0698	Dihydroxy-octadecadienoic acid	Fatty acids
64	43.55	[M + H]^+^	438.34978	439.35744	0.9	C_30_H_46_O_2_	439.3556,393.3476,203.1779,191.1774,189.1617	3-Oxolup-20 (29)-en-28-aL	Triterpenoids
65	43.57	[M + H]^+^	456.36035	457.36785	0.5	C_30_H_48_O_3_	457.2337,411.3602,297.2544,203.1785,189.1635,121.1007	Ursolic acid	Triterpenoids
66	43.93	[M–H]^−^	340.24023	339.23292	0.9	C_23_H_32_O_2_	339.2330,163.1155	Dimethisterone	Steroid
67	44.15	[M–H]^−^	280.24023	279.23385	3.2	C_18_H_32_O_2_	279.2334,261.2223	Linoleic acid	Fatty acids
68	44.62	[M + H]^+^	454.3447	455.35225	0.6	C_30_H_46_O_3_	455.3163,437.3401,409.3455,329.2449,283.2401,203.1777,189.1626	Oleanonic acid	Triterpenoids
69	44.62	[M + H]^+^	436.33413	437.3416	0.4	C_30_H_44_O_2_	437.3410,391.3345,215.1770,203.1785,189.1626,133.1000	Ursa-2,9 (11),12-trien-24-oic acid	Triterpenoids
70	45.14	[M + H]^+^	428.36543	429.37266	−0.1	C_29_H_48_O_2_	429.3692,411.3598,393.3497,357.3497,175.1106	5α-stigmastan-3,6-dione	Steroid
71	45.48	[M + H]^+^	278.22458	279.23161	−0.9	C_18_H_30_O_2_	279.0936,201.0436,149.0213,121.0999	Estrane-3,17-diol	Steroid
72	46.86	[M–H]^−^	284.27153	283.26528	3.6	C_18_H_36_O_2_	283.2648,265.2529	Stearic acid	Fatty acids
73	48.00	[M–H]^−^	576.43899	575.43149	−0.4	C_35_H_60_O_6_	575.4606,557.4487,295.2381,241.2207	β-daucosterin	Steroid

### Amelioration of Body Weight Loss and Arthritis Score in Adjuvant-Induced Arthritis Rats by *P. heterophyllum*

**FIGURE 3:**
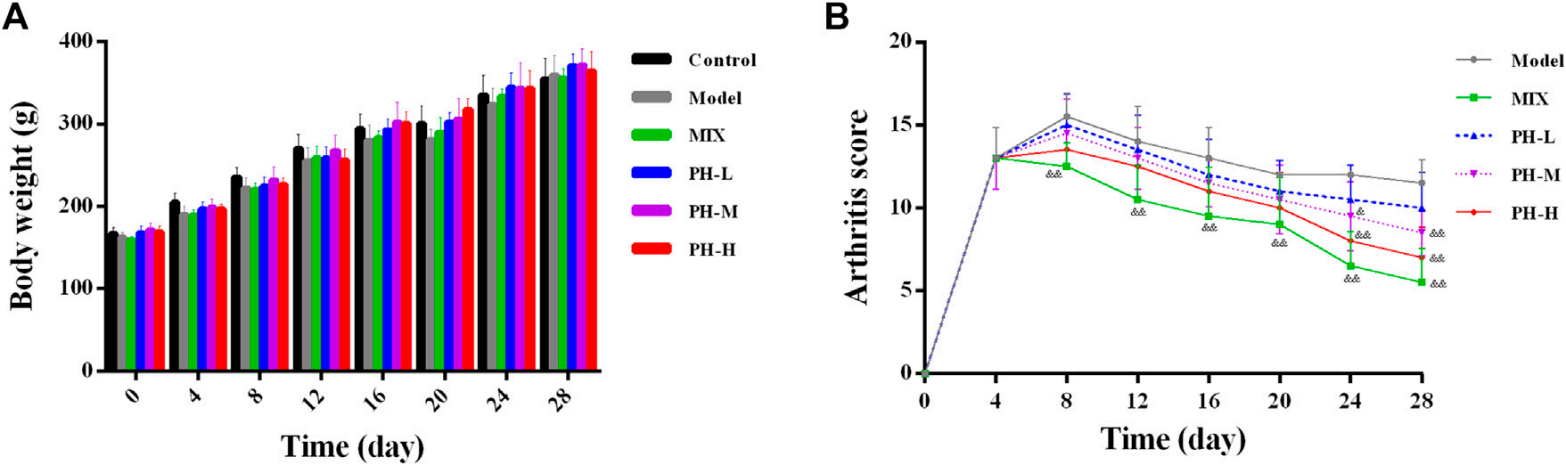
Effect of *P. heterophyllum* on the body weight **(A)** and arthritis score **(B)** in the AIA rats. Data are shown as mean ± SD (n = 8). Differences were analyzed using ANOVA by Tukey’s test. ^&^ *p* < 0.05 and ^&&^ *p* < 0.01 compared with the model group.

The body weight and arthritis score of the rats in this experiment were evaluated at 4-day intervals from day 0 to day 28. As shown in Figure 3A, the body weight of the normal control rats increased steadily throughout the process, whereas the body weight slowly increased in AIA model rats. Importantly, *P. heterophyllum* treatment in three doses (160, 320, and 640 mg/kg) ameliorated the body weight loss of the model rats to some extent.

As presented in Figure 3B, the rats in the model group had markedly higher arthritis scores compared to the normal control group (arthritis scores = 0, *p* < 0.01). After drug treatment, the positive drug methotrexate (MTX) showed prominently decreased arthritis scores compared to the model group from day 8 (*p* < 0.01). Similar to the MTX treatment, after administration of PH-M (320 mg/kg) and PH-H (640 mg/kg), the arthritis scores values decreased significantly from day 24 (*p* < 0.01 or *p* < 0.05). These results indicate that *P. heterophyllum* possesses a potent anti-RA effect in AIA model rats.

### Improving the Histopathology Lesions in Adjuvant-Induced Arthritis Rats by *P. heterophyllum*

**FIGURE 4:**
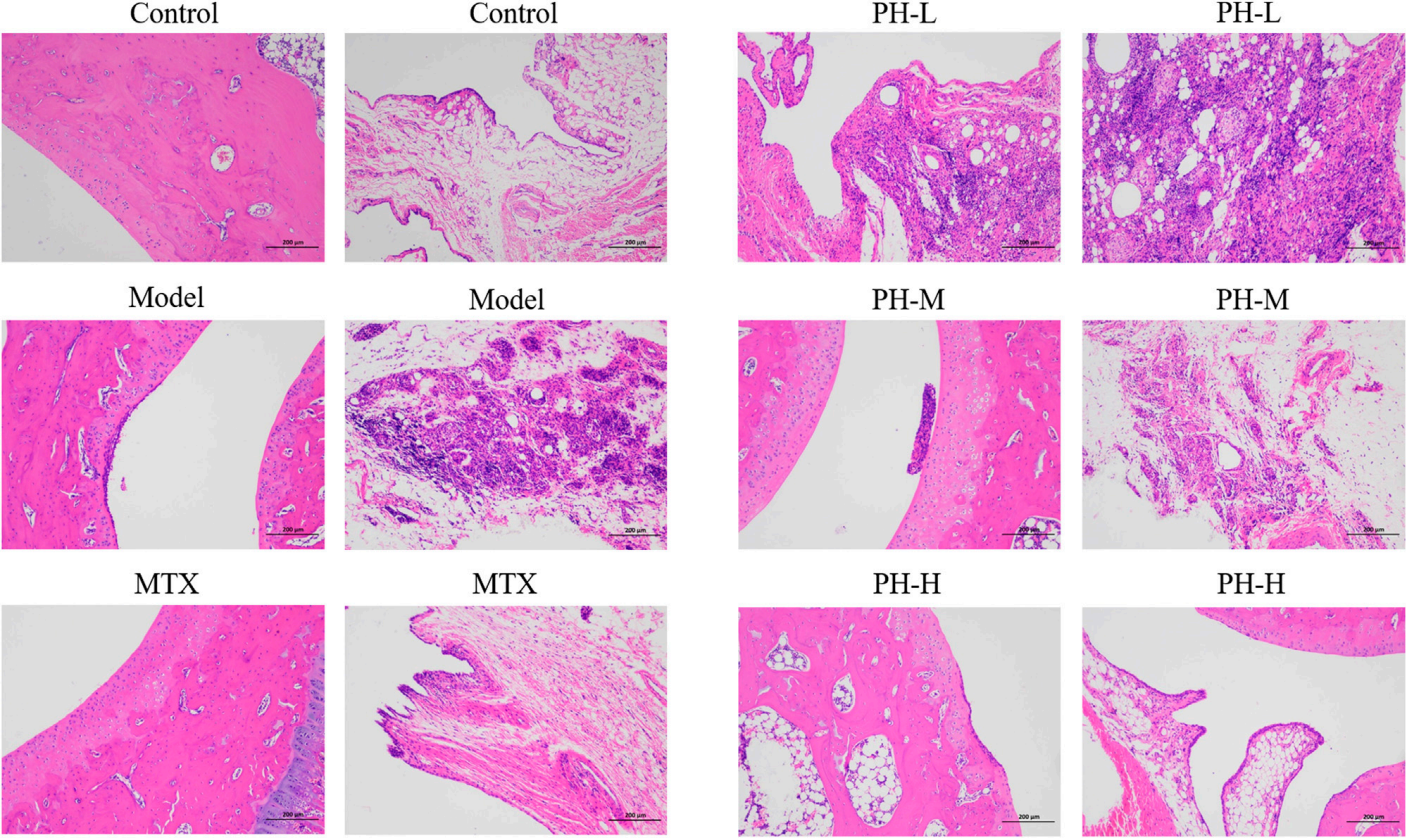
Effect of *P. heterophyllum* on histopathological changes of ankle joints in the AIA rats (×100, HE staining).

Histopathological examination is the most informative and intuitive technique for exploring the manifestations of RA disease. Compared to the normal control rats, histopathological changes of the ankle joint in AIA model rats were characterized by massive inflammatory cell infiltration into synovial tissue, pannus formation, synovial hyperplasia, and bone and cartilage erosions (Figure 4). These abnormal histopathological changes were prominently alleviated in AIA model rats after treatment with MTX and *P. heterophyllum*, especially *P. heterophyllum* at a dosage of 640 mg/kg.

### Decrease in Thymus and Spleen Indices in Adjuvant-Induced Arthritis Rats by *P. heterophyllum*

**FIGURE 5:**
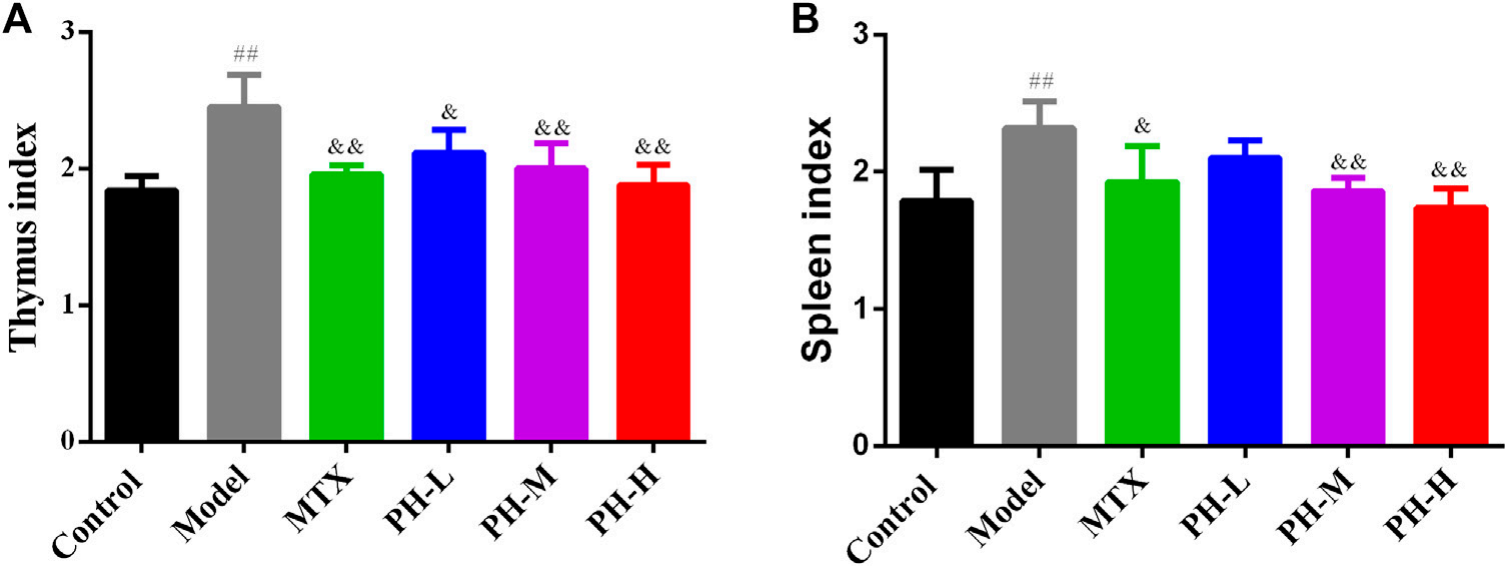
Effect of *P. heterophyllum* on the indexes of thymus **(A)** and spleen **(B)** of AIA rats. Data are shown as mean ± SD (n = 8). Differences were analyzed using ANOVA by Tukey’s test. ^##^ *p* < 0.01 compared with the control group, ^&^ *p* < 0.05 and ^&&^ *p* < 0.01 compared with the model group.

The results summarized in Figure 5 indicate that the weights of the thymus (Figure 5A) and spleen (Figure 5B) increased remarkably in the rats of the AIA model group in contrast to the rats of the normal control group (*p* < 0.01). After treatment with three crude extracts of *P. heterophyllum* and MTX, the weight of the thymus and spleen persuasively decreased (*p* < 0.01 or *p* < 0.05) compared to the model group. The results showed that rats in the AIA model group had hyperimmune response after intradermal injection of CFA, while *P. heterophyllum* could suppress abnormal immune function.

### Decreasing Serum Levels of Rheumatoid Factor and C-Reactive Protein in Adjuvant-Induced Arthritis Rats by *P. heterophyllum*

**FIGURE 6:**
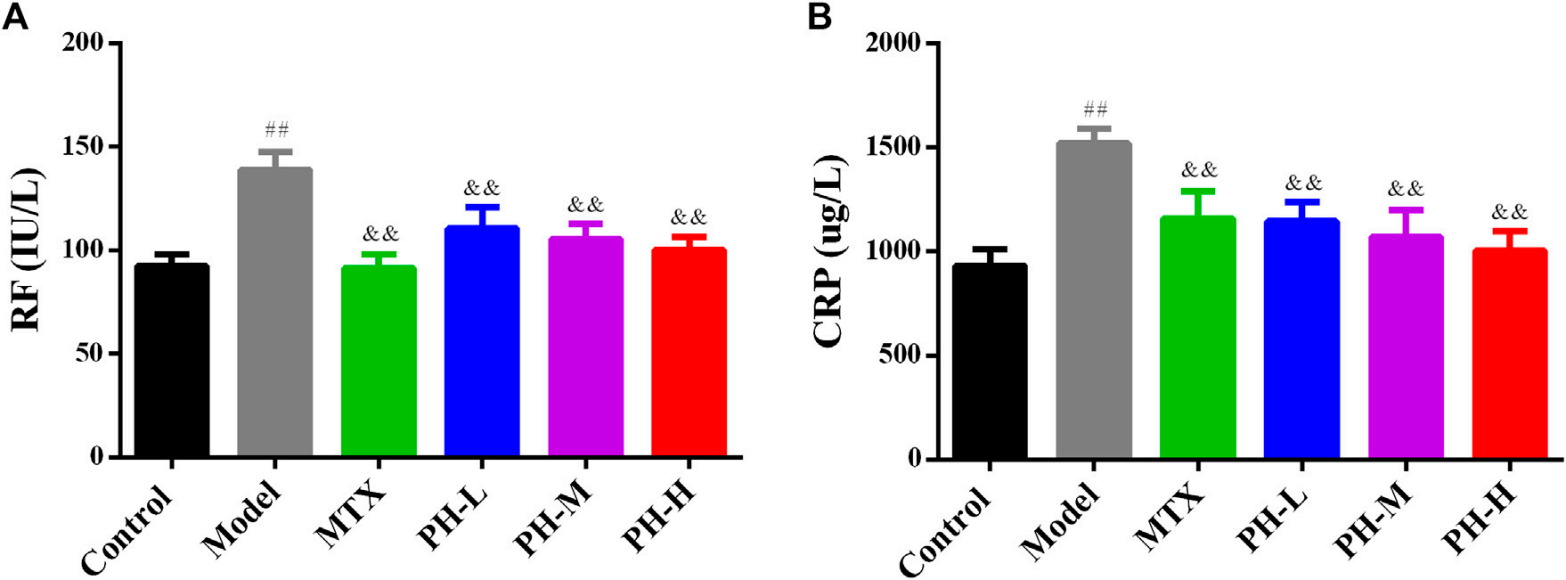
Effect of *P. heterophyllum* on the serum levels of RF **(A)** and CRP **(B)** in AIA rats. Data are shown as mean ± SD (n = 8). Differences were analyzed using ANOVA by Tukey’s test. ^##^ *p* < 0.01 compared with the control group, ^&&^ *p* < 0.01 compared with the model group.

As summarized in Figure 6, the serum levels of RF and CRP in AIA model rats were significantly higher than those of rats in normal control group (*p* < 0.01). The three crude extracts from treatment with *P. heterophyllum* and MTX observably downregulated the levels of RF and CRP in serum (*p* < 0.01).

### Decreasing Serum Levels of Pro-inflammatory Cytokines in Adjuvant-Induced Arthritis Rats by *P. heterophyllum*

**FIGURE 7:**
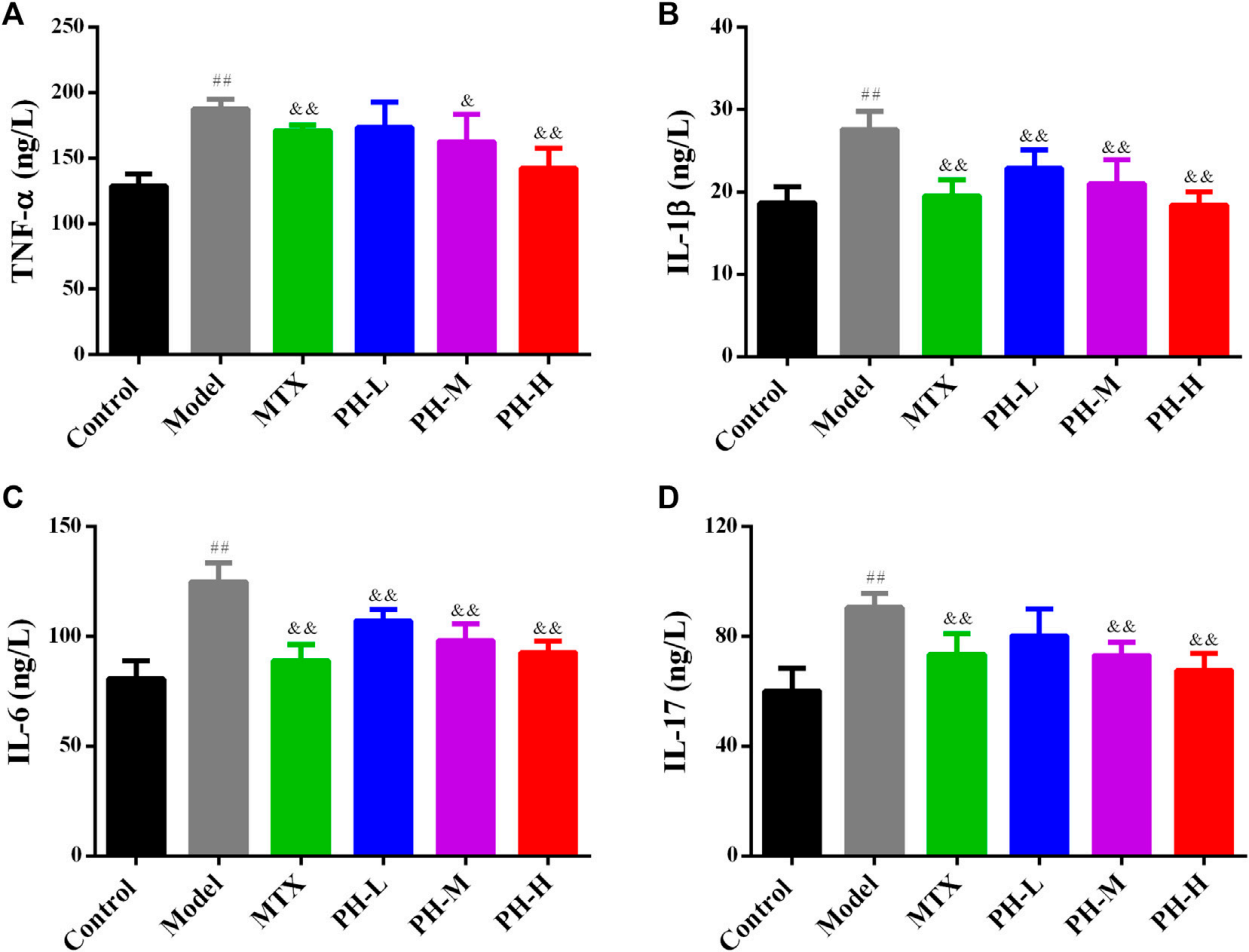
Effect of *P. heterophyllum* on the serum levels of TNF-α **(A)**, IL-1β **(B)**, IL-6 **(C)** and IL-17 **(D)** in AIA rats. Data are shown as mean ± SD (n = 8). Differences were analyzed using ANOVA by Tukey’s test. ^##^ *p* < 0.01 compared with the control group, ^&^ *p* < 0.05 and ^&&^ *p* < 0.01 compared with the model group.

The results showed that serum concentrations of TNF-α, IL-1β, IL-6 and IL-17 increased prominently (*p* < 0.01) in the AIA model group compared to the normal control group. Treatment with *P. heterophyllum* markedly decreased (*p* < 0.01) the serum levels of all the above-mentioned anti-inflammatory cytokines (Figure 7).

### Increasing Serum Levels of Anti-inflammatory Cytokines in Adjuvant-Induced Arthritis Rats by *P. heterophyllum*

**FIGURE 8:**
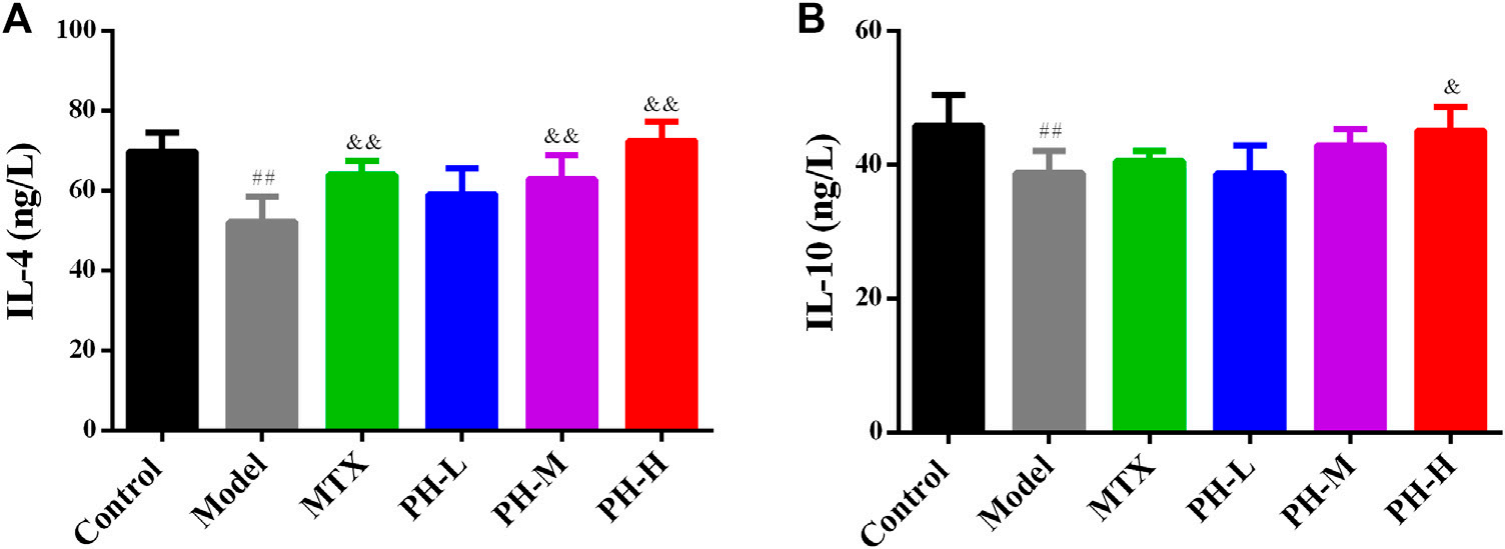
Effect of *P. heterophyllum* on the serum levels of IL-4 **(A)** and IL-10 **(B)** in AIA rats. Data are shown as mean ± SD (n = 8). Differences were analyzed using ANOVA by Tukey’s test. ^##^ *p* < 0.01 compared with the control group, ^&^ *p* < 0.05 and ^&&^ *p* < 0.01 compared with the model group.

Compared to rats in the normal control group, the levels of anti-inflammatory cytokines including IL-4 and IL-10 in the serum of AIA model group rats were significantly up-regulated (*p* < 0.01, Figure 8). Treatment with 640 mg/kg of *P. heterophyllum* remarkably down-regulated the levels of IL-4 and IL-10 in the serum of AIA model rats.

### Decreasing Peripheral Blood Mononuclear Cells Levels of Cyclooxygenase-2, 5-Lipoxygenase and Matrix Metalloproteinase-2 in Adjuvant-Induced Arthritis Rats by *P. heterophyllum*

**FIGURE 9:**
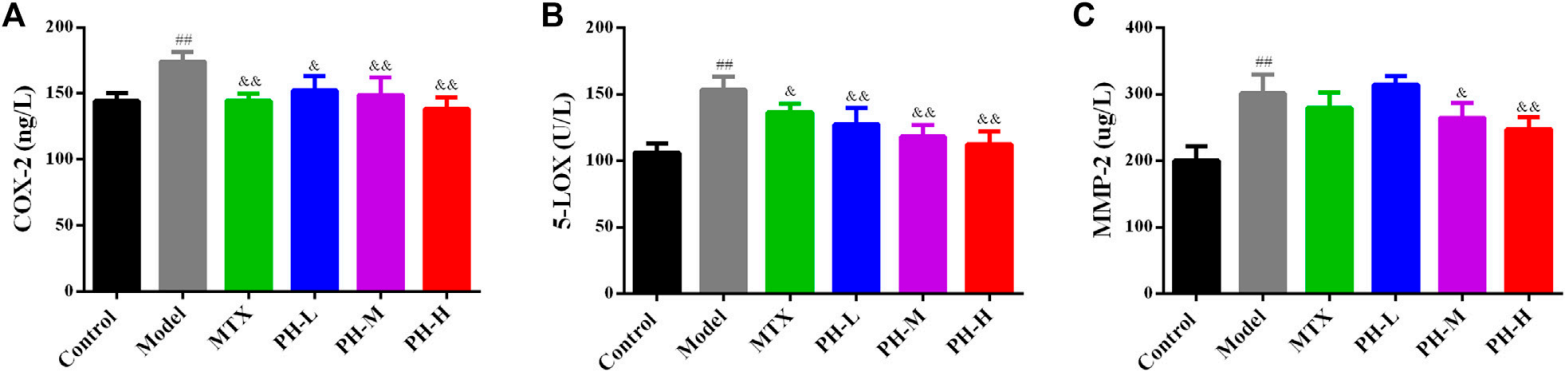
Effect of *P. heterophyllum* on the PBMC levels of COX-2 **(A)**, 5-LOX **(B)** and MMP-2 **(C)** in AIA rats. Data are shown as mean ± SD (n = 8). Differences were analyzed using ANOVA by Tukey’s test. ^##^ *p* < 0.01 compared with the control group, ^&^ *p* < 0.05 and ^&&^ *p* < 0.01 compared with the model group.

The levels of inflammatory mediators (COX-2 and 5-LOX) and MMP-2 in the rat PBMC were also evaluated by ELISA kits (Figure 9). The results showed that the levels of COX-2, 5-LOX and MMP-2 in PBMC of model rats were remarkably reduced than those of normal control rats (*p* < 0.01). After treatment with *P. heterophyllum* and MTX, the levels of COX-2, 5-LOX and MMP-2 were significantly elevated (*p* < 0.01 or *p* < 0.05) compared to those of the model group.

## Discussion

RA is the most prevalent chronic and long-term autoimmune inflammatory disease (Wang et al., 2017a; Li et al., 2019a; Saleem et al., 2020; Zhu et al., 2020). Although there are many anti-RA drugs in clinic, such as immunosuppressants, biological agents, DMARDs, steroidal drugs, and NSAIDs, most of them are associated with long-term adverse effects and costs (Li et al., 2019b; Dai et al., 2020; Zhu et al., 2020). In addition, the CFA-induced arthritis (AIA) model and the collagen-induced arthritis model are two typical preclinical experimental animal models of RA, and the former is a classic, easy-to-measure, short duration, reliable and reproducible test animal model, which is extensively used for the preclinical evaluation of anti-RA drugs since its pathological and morphological characteristics were similar to those of human RA (Yang et al., 2016; Pan et al., 2017; Voon et al., 2017; Zhang et al., 2019; Saleem et al., 2020).

The roots of *P. heterophyllum* have been widely used to treat RA as a vital TCM for centuries (Editorial Committee of Traditional Chinese Medicine 1999; Yang et al., 2016), but their anti-RA effect and chemical profiling have not been reported so far. Previous phytochemical studies have found that only 42 secondary metabolites, including six phenylpropanoids, eight triterpenoids, four flavonoids, 14 phenols and 10 others, were reported. In parallel, only 5-hydroxy-2-methoxy-1,4-naphtoquinone and taraxer-14-ene-1α,3β-diol exhibited antitumor effects *in vitro* (Yang et al., 2019a). In this work, we reported for the first time the anti-RA effect and chemical profiling of *P. heterophyllum*, thereby identifying 34 flavonoids and 39 others, while 15 flavonoids, including procyanidin B2, dihydromyricetin (-)-epicatechin, puerarin, rutin, naringin, hesperidin, myricetin, eriodictyol, quercetin, naringenin, kaempferol, diosmetin, nobiletin, and tangeretin, have been reported to have anti-RA effects in rats. Consequently, flavonoids may be responsible for the major active constituents in the roots of *P. heterophyllum* against RA as traditional folk medicine in China for centuries; however, further studies are needed to isolate and identify the bio-constituents directly related to anti-RA activity and its probable mechanism *in vivo* and *in vitro* of this plant.

In the animal model of AIA, histopathological lesions were aggravated due to massive inflammatory cell infiltration into synovial tissue, synovial hyperplasia, pannus formation, and bone and cartilage erosion (Lin et al., 2013; Voon et al., 2017; Rui et al., 2019; Yang et al., 2020). In the present research, *P. heterophyllum* exhibited the possible anti-RA effect, which prominently alleviates the above-mentioned abnormal histopathological changes in AIA model rats, accompanied by the reduction of inflammatory cytokines. Moreover, there is a straightforward relationship between weight loss/slow gain in rats and the massive inflammatory cell infiltration into synovial tissue (Lin et al., 2013; Pan et al., 2017; Jing et al., 2019). In this study, with *P. heterophyllum* treatment, body weight rose continuously in AIA model rats compared to rats in the model group. In addition, the arthritis score is a vital index to measure the anti-RA effect of drugs (Lin et al., 2013; Pan et al., 2017; Saleem et al., 2020) and is employed here to evaluate the possible therapeutic effect of *P. heterophyllum*, which was significantly decreased from day 24 compared to the model group. Finally, the spleen and thymus are two important immune organs, and their hyperfunction is closely related to the stimulation of the immune system in the AIA model rat (Lin et al., 2013; Zuo et al., 2018; Xiong et al., 2019), and the simultaneous decrease of the thymus and spleen indices by *P. heterophyllum* indicate the conceivable immunosuppressive effect.

In RA, serum RF and CRP are considered to be two important biomarkers of systemic inflammation in RA, indicating an active inflammatory response and are used to assess arthritic activity in rats with RA (Arjumand et al., 2019). This study shows that the expression of RF and CRP in serum of AIA model rat is remarkably increased, and the significant deduction after treatment with *P. heterophyllum* also suggests the feasible immunosuppressive activity.

A large number of studies have demonstrated that inflammation is a primary mechanism and a crucial role in rats with RA (Jing et al., 2019; Li et al., 2019c; Lin et al., 2013; Pan et al., 2017). Moreover, infiltration of pro-inflammatory cytokines such as TNF-α, IL-1β, IL-6 and IL-17, inflammatory mediators augment like COX-2 and 5-LOX, reduction of anti-inflammatory factors such as IL-4 and IL-10, which have been positively related to RA, causes synovial inflammation and cartilage damage (Jing et al., 2019; Li et al., 2019c; Lin et al., 2013; Pan et al., 2017). In RA, TNF-α, IL-1β, IL-6 and IL-17 play a decisive and synergistic role in synovial inflammation and cartilage damage (Jing et al., 2019; Yu et al., 2019; Saleem et al., 2020). In addition, the overproduction of TNF-α elevates the levels of IL-1β and IL-6, and generates matrix degrading enzymes (Jing et al., 2019). Likewise, IL-1β promotes osteoclast activation and MMP generation, just like increasing the expression of MMP-1, which ultimately leads to bone injury (Jing et al., 2019). On the other hand, IL-6 incites immunological reaction, MMP overproduction, and osteoclast differentiation and formation (Jing et al., 2019). IL-17 also plays a pivotal role in RA, which promotes the overproduction of pro-inflammatory cytokines and MMPs, as well as the activation of the osteoclasts and angiogenesis (Jing et al., 2019). Based on the above, therapeutic substances that particularly impede the production of TNF-α, IL-1β, IL-6 and IL-17 distinguish a crucial target for RA treatment (Jing et al., 2019; Rui et al., 2019; Yu et al., 2019). IL-4 and IL-10 by contrast, are two pivotal anti-inflammatory cytokines, which also play an important role in regulating the levels of endogenous pro-inflammatory cytokines during RA (Jing et al., 2019; Saleem et al., 2020). Our results indicate that treatment of *P. heterophyllum* obviously reduces the levels of TNF-α, IL-1β, IL-6 and IL-17, and increases the expression of IL-4 and IL-10, implying that the anti-RA effect of *P. heterophyllum* is achieved to a certain extent via the inhibition of pro-inflammatory cytokines and the elevation of anti-inflammatory cytokines in AIA model rats.

COX-2 is an overexpression of inflammatory tissues such as rheumatoid disease, and is a pivotal enzyme involved in the production of pro-inflammatory cytokines and cartilage destruction (Lin et al., 2013, Lin et al., 2014; Jing et al., 2019; Rui et al., 2019). Moreover, 5-LOX is the decisive enzyme involved in the synthesis of leukotriene, which is directly responsible for RA diseases (Lin et al., 2013, Lin et al., 2014). In addition, MMPs belong to the family of proteolytic enzymes, which play a crucial role during RA and are primarily responsible for bone and cartilage erosion (Jing et al., 2019; Yu et al., 2019). Our results demonstrate that PBMC levels of COX-2, 5-LOX and MMP-2 are highly expressed in AIA model rats, while a significant decrease is observed in PH-treated rats.

## Conclusion

In summary, the chemical profiling and anti-RA effects of *P. heterophyllum* on AIA in rats were studied for the first time. The results demonstrate that flavonoids may be partly responsible for the major anti-RA effect of *P. heterophyllum*, which can ameliorate joint damage and suppress the hyperimmune response via downregulation of pro-inflammatory cytokines (TNF-α, IL-1β, IL-6 and IL-17), inflammatory mediators (COX-2 and 5-LOX) and MMP-2, and upregulation of anti-inflammatory cytokines (IL-4 and IL-10). Our findings suggest that *P. heterophyllum* possesses the therapeutic effect of RA and supports the claim that it is a vital folk medicine in TCM for treating RA and inflammation-related diseases for centuries.

## Data Availability Statement

The raw data supporting the conclusions of this article will be made available by the authors, without undue reservation.

## Ethics Statement

The animal study was reviewed and approved by JZLLSC2018-0701.

## Author Contributions

JH designed the project and wrote the manuscript. LY, JH, RL, AF, JZ and YZ performed the experiments and analyzed the data. JH and LY discussed the data.

## Funding

This work was financially supported by the National Natural Science Foundation of China (NSFC) (No. 81760705), the Natural Science Foundation of Jiangxi Province (Nos. 20192BBHL80008 and 20192BAB215059), and the Jiangxi University of Traditional Chinese Medicine (no. JXSYLXK-ZHYA0031).

## Conflict of Interest

The authors declare that the research was conducted in the absence of any commercial or financial relationships that could be construed as a potential conflict of interest.
