# Supplementary material for: Chemical Composition of Pterospermum heterophyllum Root and its Anti-Arthritis Effect on Adjuvant-Induced Arthritis in Rats via Modulation of Inflammatory Responses
Source: Front Pharmacol. 2020 Dec 11;11:584849. doi: 10.3389/fphar.2020.584849 (PMC7759541; doi:10.3389/fphar.2020.584849)
Supplement: Supplementary file 1 [file image1.pdf]

Supplementary Material

Chemical Composition of *Pterospermum heterophyllum* Root and Its Anti-Arthritis Effect on Adjuvant-Induced Arthritis in Rats *via* Modulation of Inflammatory Responses

Li Yang[1], Ronghua Liu[1], Aiguo Fan, Jingjing Zhao[2], Yong Zhang[1] and Junwei He[1*]

*** Correspondence:** Junwei He: hjwjn2008@163.com

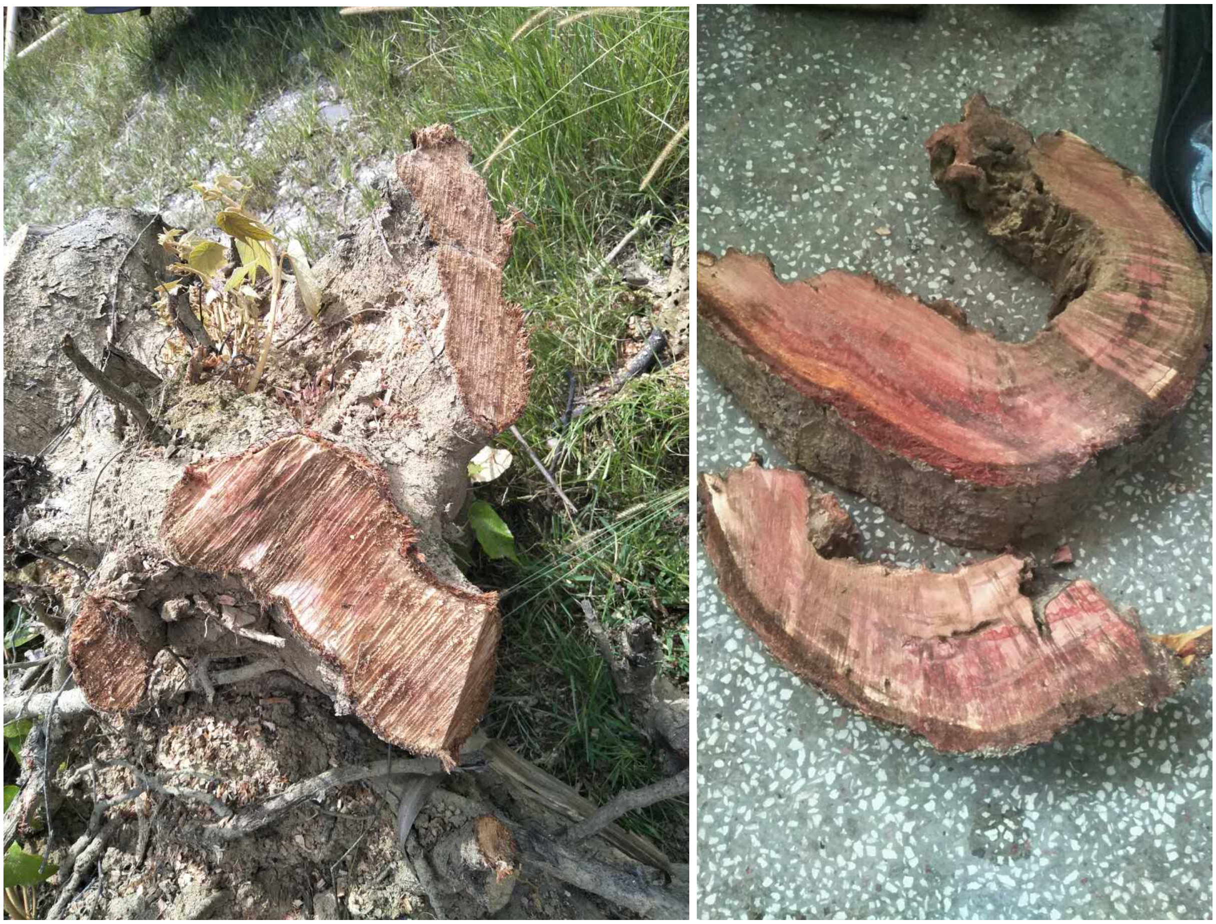

Supplementary Figure 1. The roots of *Pterospermum heterophyllum*.
